# The Impact of Graphene Oxide Nanoparticles Decorated with Silver Nanoparticles (GrO/AgNP) on the Cellulose Acetate (CA) Membrane Matrix Used for Hydrocarbon Removal from Water

**DOI:** 10.3390/membranes15060158

**Published:** 2025-05-23

**Authors:** Marian Băjan, Diana Luciana Cursaru, Sonia Mihai

**Affiliations:** Faculty of Petroleum Refining and Petrochemistry, Petroleum-Gas University of Ploiesti, 39 Bucharest Blvd., 100680 Ploiesti, Romania; marian.bajan@upg-ploiesti.ro (M.B.); smihai@upg-ploiesti.ro (S.M.)

**Keywords:** cellulose acetate membranes, decorated graphene oxide nanoparticles, silver nanoparticles

## Abstract

Adding nanomaterials to polymer membranes can improve certain properties, such as the photocatalytic degradation of contaminants and antibacterial qualities. However, the interaction between nanomaterials and polymers is often limited by the presence of functional groups that can trap nanostructures within the polymer matrix. This study focuses on the synthesis of silver-decorated graphene oxide nanoparticles and their integration into cellulose acetate membranes. Characterization of the membranes was conducted using various techniques, including electron microscopy (SEM), thermogravimetric analysis, FTIR, goniometry, and filterability tests. The results indicate that CA membranes with decorated nanoparticles exhibit improved thermal stability, making them more effective for removing heavy hydrocarbons without the risk of nanomaterial elution during temperature fluctuations in the contaminated water flow subjected to filtration. Furthermore, these decorated structures enhance hydrophobicity due to interactions between the oxygenated groups of GrO and silver ions. While these additional networks may reduce the permeate flow rate, they significantly increase the efficiency of contaminant removal.

## 1. Introduction

Global water consumption is steadily rising due to population growth and improved living conditions. However, water contaminated with hydrocarbons poses a significant risk to human health and leads to the degradation of ecosystems. This contamination often results from hydrocarbon extraction, refining processes, or household discharges into municipal wastewater treatment systems [1,2,3].

Oily water is typically found in emulsions stabilized by surfactants, making it challenging to separate using conventional wastewater treatment technologies. The adoption of third-generation filtration systems, such as membranes, can provide a high level of oily water purification while requiring minimal investment and low energy consumption [4,5,6].

The incorporation of nanomaterials into mixed matrix structures can help overcome these limitations [7,8,9,10].

Nanomaterials can serve various functional roles, including photocatalytic and antibacterial purposes. However, the compatibility between the polymeric material of the membrane and the nanoparticles is often insufficient, making it essential to identify strategies for effectively immobilizing nanostructures within the polymeric matrix [11,12].

These approaches are diverse and involve the use of highly functionalized secondary polymers to establish additional interactions between the polymer matrix and the nanomaterials. This can include the functionalization of nanomaterials with polymers or, as studied in the current work, the use of decorated nanostructures to adjust hydrophilicity [13].

Highly functionalized polymers can be integrated into mixed matrix membrane formulations, bridging the functional groups of the organic materials from which they are derived and the metal phase intended for immobilization. A previous study demonstrated that using functionalized m-phenyldiamine (mPDAf) increases the compatibility between CA and zinc oxide (ZnO) nanoparticles [14].

The functionalization of nanomaterial surfaces has been extensively studied due to its versatility and simplicity. By applying a polymer film to the surface of nanoparticles (NPs), interactions with the polymer matrix are modified, leading to changes in their properties. These characteristics can be tailored by selecting the appropriate nanomaterial, polymer, or synthesis method. The attachment of organic groups to the nanoparticle surface occurs through metal–carbon covalent bonds. Coating nanomaterials with polymers that have a high degree of crosslinking and contain functional groups, such as hydroxyl (-OH), carboxyl (-COOH), amino (-NH_2_), and thiol (-SH), enhances the adhesion between inorganic nanoparticles and the polymer [15,16].

The application of a polydopamine (PDA) film serves as a bonding platform between materials with different functionalities. The immobilization of metal particles within polydopamine to create metal–polymer nanocomposites occurs via catechol groups. Research has shown that in an alkaline medium, polydopamine undergoes self-polymerization reactions, resulting in the formation of microspherical particles with dimensions of approximately 145 nm. These particles are then dispersed in nanoparticle solutions through ultrasonication [17].

In the case of PDA–silver assembly, it has been reported that stretching vibrations of the NO group appear at a wave number of 1580 cm^−1^, a phenomenon not observed in simple PDA. By oxidizing catechol and reducing silver ions, carbonyl and quinone products are generated, which enhance the interaction between the nanoparticles and PDA, thereby preventing nanoparticle aggregation [18,19].

Another polymer commonly used to coat nanoparticles is polyethylene glycol (PEG, C_2n_H_4n+2_O_n+1_). PEG serves as a chelating agent and utilizes the hydroxyl groups located at the periphery of its chain to create a bridge between compounds with different polarities, such as polymers and metals or polymers and metal oxides. Coating nanoparticles with a thin layer of PEG helps prevent their agglomeration and mitigates the effects of electrostatic repulsion [20,21].

Additionally, the use of polyethyleneimine (PEI, (C_2_H_5_N)_n_) facilitates the stabilization of nanoparticles through its amino groups, allowing for the formation of covalent bonds. It can be concluded that the interactions between nanometric structures and their polymer coatings depend on the functional groups present in the polymers. To effectively regulate the degree of agglomeration, it is advisable to use polymers with higher molecular weights, as they possess a greater number of coordination sites and can form stronger attachments around the nanoparticles [17,22].

The third approach studied to enhance the compatibility of nanostructures with polymers for membrane production involves the use of decorated nanomaterials. By depositing metals or metal oxides on graphene nanosheets, we can achieve products with improved electrical, optical, catalytic, and antibacterial properties. Additionally, the decoration method allows for better control over the hydrophilicity of the nanoparticles. Graphene is the most commonly used support material due to the availability of its carbonyl functional groups, which facilitate interactions with inorganic materials. The chemical structure of graphene imparts a strongly electronegative character, while the nanoparticles, which may be metals or metal oxides, typically carry positive electrical charges. This difference in electrical charge leads to strong interactions between the materials, primarily attributed to electrostatic forces [23,24,25,26].

Polymeric membranes that incorporate AgNPs are recognized for their significant potential in treating polluted water streams. These membranes exhibit exceptional antifouling and antimicrobial properties, achieving removal efficiencies of over 99.9% for bacteria such as Escherichia coli and Staphylococcus aureus. However, the long-term stability of silver nanoparticles within the membrane structure can be limited. As these filters are used in industrial applications or undergo multiple cycles of use, there is a risk of silver leaching over time, resulting from the release of silver ions or the nanoparticles themselves from the polymer matrix [27,28,29].

Several studies have reported the production of GrO through chemical exfoliation methods such as the Hummers or Tour processes. Silver is then deposited onto the surface of GrO nanosheets using sonication or mechanical agitation with a silver nitrate solution. These decorated nanostructures can be incorporated into the polymeric membrane matrix or serve as an active layer on the filtration medium’s surface [30,31,32]. In this work, silver was utilized in the form of nanometric dimensions of up to 100 nm.

Zeeshan et al. investigated the impact of GrO/AgNPs on the performance of nanofiltration membranes in rejecting Cr (VI) and Co (II) when integrated into a cellulose acetate/polyethylene glycol (CA/PEG) polymer matrix. They reported improvements in porosity and surface roughness, which were attributed to the interactions between the oxygenated functional groups of the nanomaterials and those of the polymer [33].

Sun et al. developed a coating layer made up of GrO and AgNPs applied to the surface of a polymeric membrane to control biofilm formation. Their findings highlighted the role of the hydroxyl, epoxy, and carboxyl functional groups of GrO, which serve as adsorption sites for silver ions. This interaction reduces the hydrophilicity of the membranes compared to structures that contain only graphene oxide [34].

Song et al. reported that the GrO–AgNP assembly acts as a photocatalyst by generating electron–hole pairs. The presence of silver nanoparticles increases the light absorption rate, while partially reduced GrO enhances electron mobility and delays their recombination, thereby improving photocatalytic efficiency [35].

This study focuses on the structural changes that nanoparticles undergo after decoration. The objective is to create GrO decorated with AgNPs and incorporate this into CA membranes. This integration aims to enhance the membranes’ permeability and improve their resistance to biofouling. The GrO, upon which the AgNPs are deposited, has a strong tendency to interact with the oxygenated functional groups of CA, effectively immobilizing the inorganic nanoparticles. As a result, the membranes produced will exhibit varying thermal stabilities, permeabilities, and efficiencies in contaminant removal compared to those made solely from CA or CA with AgNPs.

To evaluate the filtration performance of nanocomposite membranes, water contaminated with heavy cycle oil (HCO) from a fluidized bed catalytic cracking plant was selected. This choice was made due to the complex composition of the HCO, which contains thousands of different hydrocarbons across several classes, including paraffins, naphthenes, olefins, and polycyclic aromatic hydrocarbons. In addition, it contains various heterocompounds, such as oxygen, sulfur, and nitrogen compounds [36]. The density of the HCO is 980 kg/m^3^, which is close to that of water, and its polar characteristics—due to the presence of polycyclic aromatics—make it challenging to separate from contaminated water flows resulting from crude oil extraction or processing. The goal was to develop a nanocomposite cellulose acetate membrane that could be effectively used in industrial applications for removing hydrocarbons from water, demonstrating good efficiency under varying temperature conditions without any leaching of the functional nanomaterial over time. Thermal analyses, including TGA and DSC, will highlight that the presence of GrO nanoparticles acts as a bridge between cellulose acetate and AgNP, facilitating additional hydrogen bonding. This advancement expands the potential applications of nanocomposite polymer membranes, particularly for use in wastewater treatment plants in refineries.

## 2. Materials and Methods

### 2.1. Chemical Reagents

All reagents used in this study were purchased from Merck–Sigma-Aldrich, Darmstadt, Germany, and are of reagent grade. Graphite was utilized to obtain GrO. The following chemicals were employed for the exfoliation of graphite using the Tour method: 98% sulfuric acid (H_2_SO_4_), 85% orthophosphoric acid (H_3_PO_4_), potassium permanganate (KMnO_4_), hydrogen peroxide (H_2_O_2_) at a concentration of 3%, and 37% hydrochloric acid (HCl).

Silver nanoparticles (with a particle size of less than 100 nm, Merck–Sigma-Aldrich, Darmstadt, Germany) were used for decorating the graphene oxide nanosheets. Cellulose acetate with an average molecular weight (Mw ~ 30,000, Merck–Sigma-Aldrich, Darmstadt, Germany) served as the base raw material for the membrane matrix, while N,N-dimethylformamide (DMF, Merck–Sigma-Aldrich, Darmstadt, Germany) was used as the solvent. Dichloroethane (Merck–Sigma-Aldrich, Darmstadt, Germany) was utilized for isolating hydrocarbons from water. Heavy cycle oil (HCO), obtained from a local refinery, was used to contaminate water for filterability tests.

### 2.2. Obtaining Graphene Oxide Nanoparticles

To obtain GrO, the Tour method was employed [36]. Initially, 2.5 g of graphite was weighed and dispersed in a solution consisting of 80 mL of 98% sulfuric acid, 20 mL of water, and 20 mL of ortho-phosphoric acid. The mixture was heated on a water bath to 60 °C and maintained at this temperature for 6 h with stirring at 270 rpm.

For oxidation, 7 g of potassium permanganate was added to the mixture in four equal portions, with each portion being added at 15 min intervals after the temperature reached 60 °C. At the end of the reaction period, the mixture was cooled, resulting in a dark brown color.

Following cooling, 170 mL of 3% hydrogen peroxide was added to reduce any unreacted permanganate. The graphene oxide nanoparticles were then separated through filtration, and the GrO cake was washed with a 0.5 N hydrochloric acid solution. The solid phase was successively washed with distilled water and subjected to centrifugation. The complete removal of water was accomplished by maintaining the sample at 70 °C for 8 h.

### 2.3. Obtaining Graphene Oxide Nanoparticles Decorated with Silver Nanoparticles

The decoration of graphene oxide nanoparticles with silver nanoparticles (GrO/AgNP) was accomplished by dispersing 0.15 g of graphene oxide and 0.357 g of silver nanoparticles in 50 mL of distilled water. The mixture was stirred for 30 min at a speed of 20,000 rpm using a disperser. Once homogenization was complete, the solution was combined with 150 mL of DMF and heated to 130 °C for 2 h, with the heating flask equipped for reflux.

After cooling, the GrO/AgNP suspension was centrifuged at 5000 rpm for 30 min. The colloids were washed three times with distilled water and separated by centrifugation to remove any excess silver ions (Ag^+^). The resulting composite was then dried at 60 °C for 6 h. The aggregation of silver on the surface of GrO was confirmed through scanning electron microscopy and thermogravimetric analysis [37].

### 2.4. Obtaining Cellulose Acetate Membranes with Nanoparticles

CA membranes were produced through a phase inversion process using water as a nonsolvent. The synthesis of mixed matrix CA membranes involved dispersing AgNP and GrO/AgNP composites in dimethylformamide (DMF) solvent. This dispersion was achieved by mechanically stirring the mixture for 30 min at a speed of 500 rpm, after which CA granules were added. Once the granules were fully dissolved, the resulting gel was allowed to sit for 24 h at 20 °C to eliminate any air bubbles [38].

The prepared gels were then poured onto the surface of a glass plate and evenly spread out using centrifugal force. The solidification of the membranes occurred by immersing them in distilled water. Details on the composition of the synthesized membranes are provided in Table 1.

### 2.5. Sample Characterization

For the qualitative analysis of the materials, Fourier-transform infrared (FTIR) spectroscopy was used to identify the functional groups present in the structures. The analysis was conducted using a Shimadzu IRAffinity-1S spectrophotometer (Kyoto, Japan), which was equipped with the GladiATR-10 accessory. The measurements were taken within the wavelength range of 380 to 4000 cm^−1^, with a spectral resolution of 4 cm^−1^.

Thermogravimetric analyses (TGA) of the materials were conducted using a Labsys Evo apparatus from Setaram (Caluire, France). The analysis was performed in a nitrogen atmosphere, covering a temperature range of 20–900 °C with a heating rate of 5 °C/min.

Differential scanning calorimetry (DSC) was performed to investigate temperature-induced transitions, such as melting and the formation of a glassy state. The analysis was conducted using a Thermal Analysis System DSC 3+ from Mettler Toledo (Greifensee, Switzerland). The samples were heated from room temperature to 400 °C at a rate of 10 °C per minute in a nitrogen atmosphere.

The goniometric analysis (GA) measured the contact angle formed between a water droplet and the membrane surfaces. A 10 µL drop of distilled water was placed on the sample surface, and its image was captured using the Intel Play Digital Microscope camera (Shenzhen, China). The contact angle of the water droplet provides information about the hydrophilicity of the samples.

The microstructural morphologies were analyzed using a scanning electron microscope (SEM) on the Scios 2 HIVAC Dual-Beam ultra-high-resolution FIB-SEM (Brno, Czech Republic) apparatus. The samples were placed on a stub with the aid of double-sided adhesive carbon conductive tape. First, the adhesive tape was applied to the stub, and then the sample was glued onto the tape. For the SEM measurements, the accelerating voltage was set to 2 kV, while for the EDS measurements, it was set to 30 kV.

The permeability test was conducted using a kit that included a 300 mL graduated funnel, a 500 mL filtrate collection container, and an aluminum clamp. A membrane was placed on the surface of a 47 mm diameter frit. A Chemker 400 PTFE pump (Taiwan) was utilized to create a vacuum, achieving a maximum pressure of −670 mmHg and a flow rate of 38 L/min.

The pollutant removal capacity was assessed using UV–vis spectrophotometry with a Shimadzu UV-1900i (Kyoto, Japan) device, which operates within a wavelength range of 190 to 1100 nm. Absorbance measurements were taken at 280 nm.

Separation of the GrO/AgNP suspension was achieved using the ROTOFIX 46 centrifuge (Târgoviște, Romania).

### 2.6. Study of Membrane Filtration Performance

To test the permeability of the membranes, an emulsion consisting of 98% water and 2% HCO was prepared. The two components formed a single phase by stirring with a disperser for 30 min at a speed of 18,000 rpm. The membrane was then placed on a ceramic frit, and a vacuum was applied to facilitate the passage of water.

#### 2.6.1. Establishing the Permeate Flow Rate

The relationship for determining the permeate flow rate was calculated as follows:

(1) $$P_{m}=\frac{V}{A_{m}*t}\left(\frac{l}{m^{2}*h}\right)$$

P_m_—permeate flow rate (l/m^2^h);

V—filtered water sample volume (l);

A_m_—membrane area (m^2^);

t—time in which the water flow passes through the unit area (h).

At the end of a filtration cycle, the surface of the membrane is washed with distilled water to eliminate any retained hydrocarbons.

#### 2.6.2. Establishing the Filtration Efficiency

After each filtration cycle, the hydrocarbon content of the permeate was measured using UV analysis at a wavelength of 280 nm. The non-polar phase of the filtrate was extracted with dichloroethane and then analyzed. The efficiency of the filtration process was evaluated by comparing the amount of hydrocarbons removed to the initial amount present. The calculation for this evaluation is as follows:

(2) $$E=\frac{c_{i}-c_{f}}{c_{i}}*100$$

E—membrane efficiency (%);

c_i_—initial concentration of the contaminant (ppm);

c_f_—concentration of the contaminant in the permeate (ppm).

## 3. Results

### 3.1. Scanning Electron Microscopy for Nanomaterials

**Figure 1:**
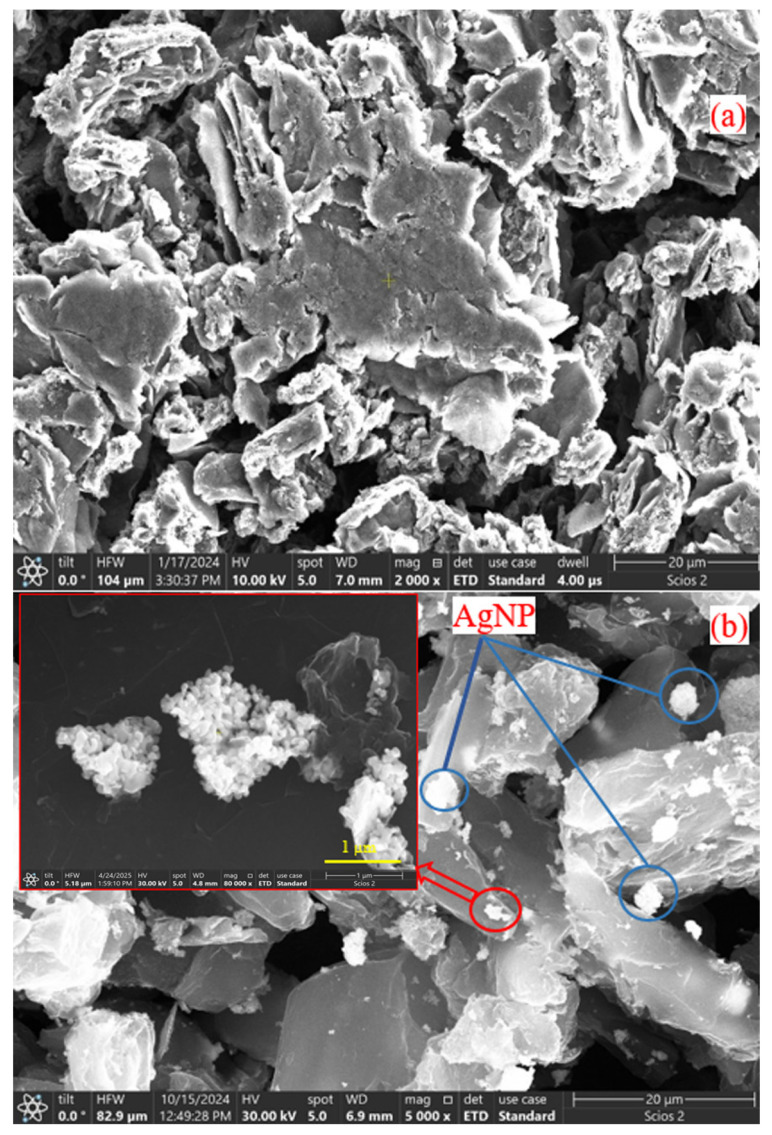
Surface morphology of graphene oxide: (**a**) before decoration; (**b**) after decoration with AgNPs.

The surface morphologies of GrO and GrO decorated with AgNPs are illustrated in Figure 1. The GrO surface exhibits wrinkled areas, which result from the presence of carboxyl and hydroxyl functional groups formed during the oxidation process. Gaps between the layers are also observed, which are attributed to the porosity of the graphene oxide nanosheets, an important characteristic for water filtration applications [39].

In image b, a structural change is evident. In addition to the aggregation of AgNPs, there is an increase in the distance between the layers due to the reduction process applied during the decoration. The size of AgNPs has been confirmed to be nanometric, with an average of 90 nm. These nanoparticles form agglomerates that attach to graphene oxide.

### 3.2. Thermogravimetric Analysis for Nanomaterials

**Figure 2:**
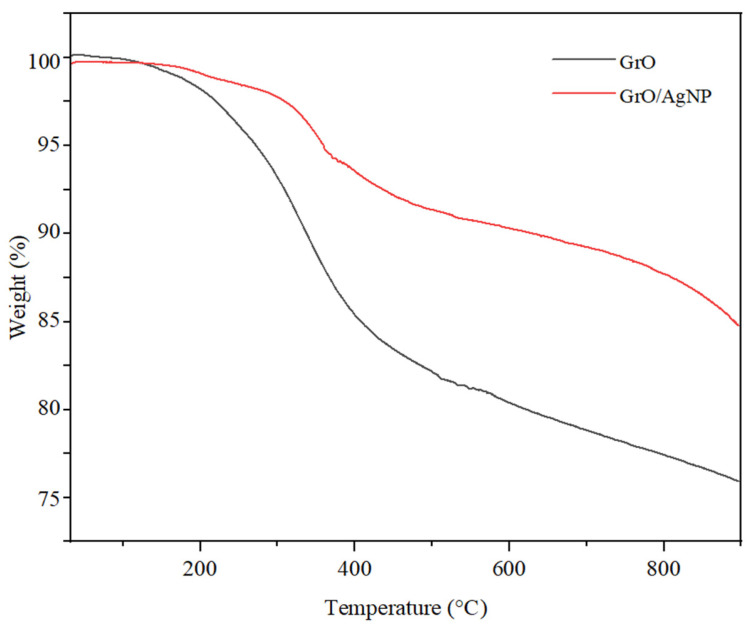
TGA profile of graphene oxide before and after decoration.

The thermal stability of graphene oxide nanosheets and graphene oxide decorated with silver nanoparticles was evaluated in a nitrogen atmosphere, with the results illustrated in Figure 2. For both types of nanoparticles analyzed, the thermogravimetric analysis (TGA) curves show an initial mass loss of 2.5% up to a temperature of 210 °C. A comparative analysis of the two structures indicates a more gradual mass loss for GrO compared to the GrO/AgNP assembly. Specifically, graphene oxide loses 18.8% of its mass by 520 °C due to the degradation of oxygenated functional groups and the breakdown of the carbon skeleton, with complete decomposition of carbon occurring by 900 °C [37,40]. In contrast, GrO/AgNP exhibits an initial mass loss at higher temperatures, which is attributed to the bonding interactions between silver and the oxygenated groups on the surface of GrO. These interactions help to inhibit the degradation of the functional groups in graphene oxide at lower temperatures. The GrO/AgNP assembly exhibits higher thermal stability, which enhances the structural stability of the membrane matrix at elevated operating temperatures within the filtration system. Additionally, this integration helps prevent the migration of AgNP and the decomposition of GrO when the membrane undergoes numerous cycles of use and is subjected to high temperatures [31].

### 3.3. FTIR Analysis for Membranes

**Figure 3:**
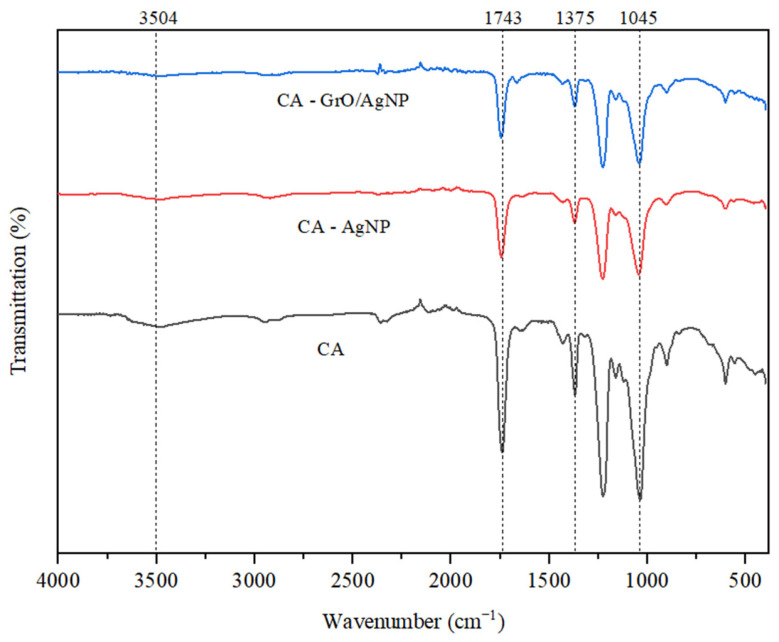
FTIR spectra of membranes.

The three membranes analyzed in this study were compared to highlight the changes occurring at the functional group level. Figure 3 displays the spectra for the CA, the CA with AgNPs, and the membrane created from cellulose acetate decorated with GrO/AgNPs. The simple CA membrane shows stretching vibrations in the range of 3520 to 3750 cm^−1^, characteristic of hydroxyl groups. The carbonyl bonds are indicated by a band appearing at 1750 cm^−1^. The strongest stretching vibrations are observed at 1240 cm^−1^ and 1030 cm^−1^, corresponding to the C-O and C-O-C groups in CA [41]. The incorporation of silver nanoparticles into the membrane matrix results in reduced transmittance due to ion–dipole interactions between the electron-rich oxygen atoms of the carboxyl groups and the electropositive cations of silver. When decorated nanomaterials are used in the membrane structure, a shift of the band towards lower wavenumber values is observed at 1743 cm^−1^. This shift is associated with electrostatic interactions and hydrogen bonding between graphene oxide and the C=O groups of CA. Additionally, changes in the bands at 1375 cm^−1^ and 1045 cm^−1^ indicate interactions between graphene oxide and the C-O-C groups [42,43].

### 3.4. DSC Analysis for Membranes

**Figure 4:**
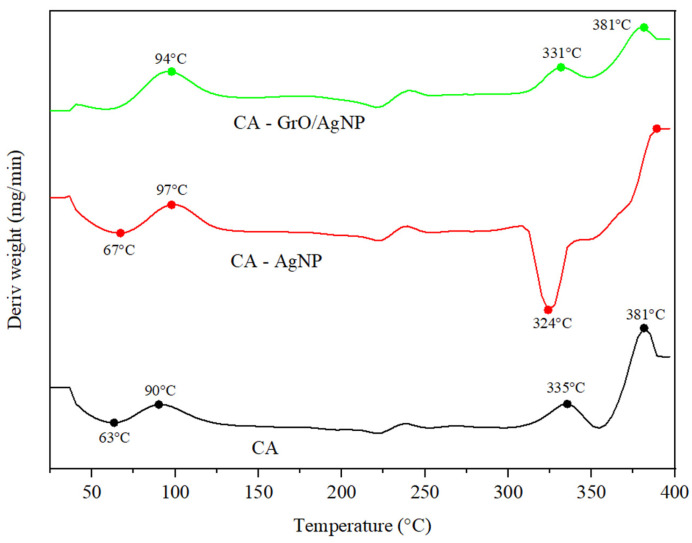
DSC profile for membranes.

The differential scanning calorimetric analysis of the three membranes is presented comparatively in Figure 4. The results indicate that both the CA and CA–AgNP membranes exhibit a broad endothermic peak in the temperature range of 25 °C to 95 °C, which corresponds to water desorption [44]. At a temperature of 324 °C, the CA–AgNP sample displays a strong endothermic peak, attributed to the presence of AgNPs, which weaken the interactions between the cellulose acetate monomers. Furthermore, the incorporation of silver deposited on the surface of GrO improves the thermal stability of the CA–GrO/AgNP assembly [45]. As a result, the endothermic peak at 324 °C disappears, and the intensity of the peak at 381 °C, observed for CA, decreases. This behavior is believed to be due to the formation of additional hydrogen bonds between the functional groups of cellulose acetate and the oxygen atoms of GrO. These findings align with the observations obtained from the FTIR analysis, which indicate an enhancement in the interactions between CA and GrO/AgNPs. The interactions between GrO/AgNPs and the polymeric material result in nanocomposite membranes that can effectively remove hydrocarbons at higher operating temperatures while maintaining their structural integrity without losing nanomaterials. This directly enhances their efficiency. Additionally, these interactions help preserve the active surface and porosity of the membranes, which is beneficial in applications where temperature fluctuations may take place [46,47].

### 3.5. TGA Analysis for Membranes

**Figure 5:**
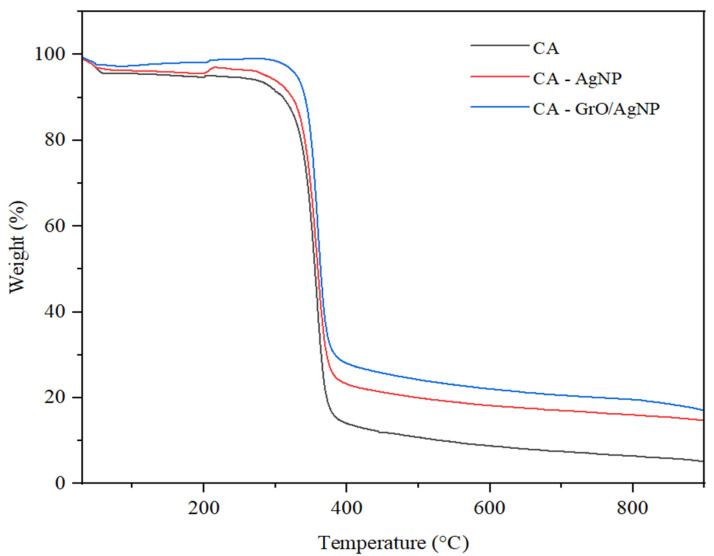
TGA profile for membranes.

The results of the thermogravimetric analyses for membranes are presented in Figure 5. Up to a temperature of 100 °C, all samples exhibit a mass loss of approximately 2%, which can be attributed to the elimination of water adsorbed in the polymer matrix. The first significant interval of thermal decomposition occurs between 295 °C and 380 °C. It is observed that the mass loss in the nanomaterial structures is lower during the initial decomposition phase. This is due to the association of nanoparticles with some oxygenated functional groups of CA [48]. The stronger interactions between CA and graphene oxide/silver nanoparticles (GrO/AgNPs) lead to the initiation of decomposition at a temperature of 324 °C. This is a result of the protective effect of hydrogen bonds formed between graphene oxide and the hydroxyl (-OH) and carboxyl (-COOH) groups of CA.

The thermal decomposition of the CA polymer is primarily due to depolymerization, the breaking of glycosidic bonds, and the occurrence of deacetylation.

The third important step of thermal degradation begins at 380 °C and continues until 900 °C. The results indicate a notable difference in the percentage of mass loss among the samples. The least stable in this temperature range is the CA membrane, which loses 24% of its mass, followed by CA–AgNP, which exhibits an 18% decrease. The better stability of CA–AgNP is attributed to the interactions between the undecomposed acetate groups and the silver ions. The increased thermal stability of the CA membrane with GrO/AgNPs indicates that the interactions between the decorated nanomaterial and the polymer have intensified. This enhancement reduces the leaching of nanostructures under high water flow pressures, numerous membrane use cycles, and varying feed temperatures. Consequently, this makes the membrane a viable solution for industrial applications, extending the time before the filtration modules need to be replaced [49,50,51].

### 3.6. SEM Analysis for Membranes

**Figure 6:**
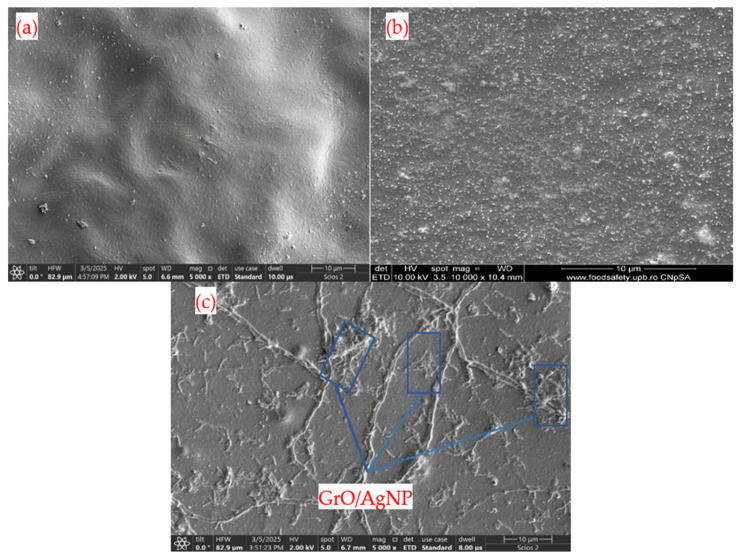
Surface morphology of membranes: (**a**) CA membrane, (**b**) CA–AgNP membrane, (**c**) CA–GrO/AgNP membrane.

The SEM image in Figure 6 shows that the CA membrane has a smooth surface characterized by a wavy shape, which is attributed to the membrane’s drying process. Additionally, there are dispersed particles present on the surface, indicating structural defects due to localized aggregations within the CA. Since the surface does not exhibit any voids, the pores are relatively small, ensuring a stable flow of water. According to Figure 6b, the presence of silver nanoparticles results in increased surface roughness. The bright dots visible in the image indicate the presence of these metallic nanoparticles. Given the nanometer scale of AgNPs, they tend to form clusters ranging from 1 to 5 µm, a trend also illustrated in Figure 1 [52]. On the other hand, the surface of the CA membrane with GrO/AgNPs displays a significantly different structure compared to the other two membranes. In this instance, networks resembling ribs are unevenly distributed across the surface, which results from the interaction between the carbon nanosheets and the polymer, leading to regions with higher particle density [53,54,55]. The interconnected nature of these ribs, along with the integration of AgNPs, enhances the immobilization of the nanoparticles. The networks created by decorated graphene oxide, as shown in Figure 6c, are lighter in color due to the presence of silver nanoparticles in their structure. Importantly, no damage to the membrane’s porosity is observed, which helps prevent the development of preferential flows that could compromise separation efficiency.

### 3.7. Goniometric Analysis for Membranes

**Figure 7:**
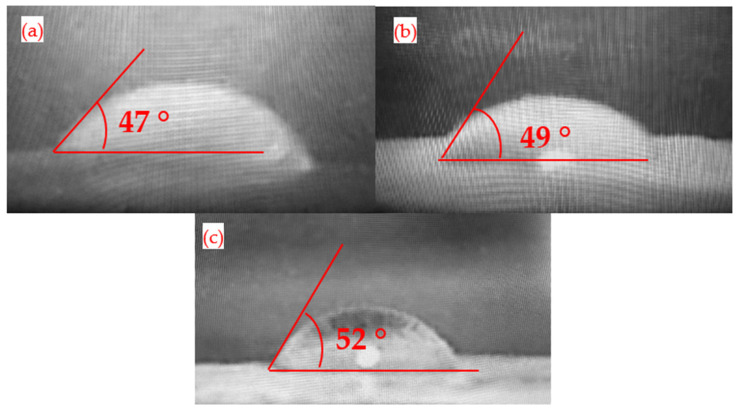
Goniometric profile for membranes: (**a**) CA mmbrane; (**b**) CA–AgNP membrane; (**c**) CA–GrO/AgNP membrane.

Figure 7 presents the results of the goniometric analysis, which compares the contact angle values of the synthesized membranes to identify variations in hydrophilicity based on their composition. A contact angle greater than 90 degrees indicates a hydrophobic character, whereas a contact angle below this value signifies hydrophilicity [56]. According to the results, all three analyzed membranes fall within the hydrophilic range, although some differences are evident. The lowest contact angle of 47 degrees was observed for the CA membrane, which is attributed to the presence of oxygenated functional groups that facilitate water transport. When AgNP are added to the composition, the contact angle increases by two degrees compared to the CA membrane. This result differs from findings reported in studies that indicate a decrease of 10 to 20 degrees in the contact angle upon incorporating silver nanoparticles [57,58]. This discrepancy can be attributed to several factors, including the hydrophobic nature of AgNPs, the high concentration used during the synthesis of the nanocomposite membranes, and their tendency to concentrate on the polymer surface during the casting process. These variables help explain the 4.2% increase in the contact angle compared to the CA membrane. The hydrophobic characteristics of silver nanoparticles are well-supported by various publications [59,60,61]. This change is likely due to the blocking of some pores in the polymer membrane by the nanoparticles and a reduction in the number of oxygenated groups in the polymer structure, as these groups are used as ligands for silver ions [62]. The most significant increase in hydrophobicity compared to the CA membrane occurs when decorated nanoparticles are used in combination. In this case, a contact angle of 52 degrees is recorded, resulting from the interactions between graphene oxide and the oxygenated functional groups of cellulose acetate.

### 3.8. Permeability Test for Membranes

This process resulted in two flows: one consisting of permeate, which has a lower concentration of the contaminant (HCO), and the other, called retentate, in which the contaminant is concentrated. In this study, each permeate flow underwent two additional successive stages of filtration through the membrane. It was observed that the presence of nanoparticles reduces the permeate flux, a trend that is further pronounced when using decorated nanoparticles in the mixed matrix of cellulose acetate polymer membranes. The molecular interactions between the functional groups of CA and the nanoparticles decrease the hydrophilicity of the membrane, which in turn reduces the flow rate [62]. Another conclusion from the permeability test is that the permeate flux is dependent on the concentration of the contaminant in the water being filtered [63].

**Figure 8:**
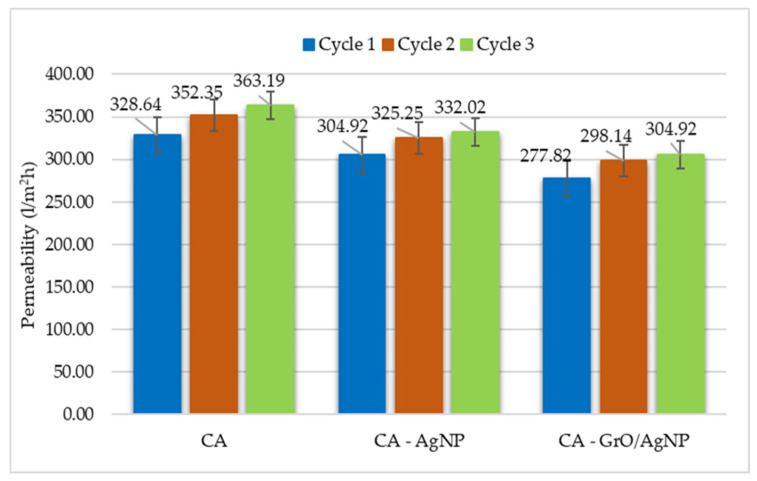
Permeability test results.

The data presented in Figure 8 for the cellulose acetate membrane demonstrate an increase in the water flow rate during the second filtration stage when utilizing the permeate. Specifically, the flow rate increases by 10.51%, changing from an initial rate of 328.64 L/m^2^h in cycle 1 to 363.19 L/m^2^h for permeate 3. Additionally, the CA–GrO/AgNP assembly shows that treatment cycle 3 has a water flow rate that is 19.11% lower compared to cycle 3 using the standard polymer membrane. Three filtration cycles were conducted using the same membrane, each at different pollutant concentrations in the feed stream. At the end of each cycle, the membrane surface was washed to remove the hydrocarbons that had accumulated in the active layer. This process eliminates the accumulated pollutants, preventing them from affecting the efficiency of the next cycle. The GrO nanosheets also contribute to enhanced hydrocarbon retention. Membranes composed of CA with AgNPs exhibit intermediate values for permeate flow rates. This behavior can be attributed to the bonds formed between the graphene oxide and the oxygenated functional groups of cellulose acetate, which enhance hydrophilicity but reduce its overall effectiveness. These findings align with the results from the goniometric analysis, which indicate that the surface of the CA membrane with GrO/AgNPs exhibits a more hydrophobic character.

The decrease in the permeate flow rate observed in the assembly containing decorated nanoparticles can be attributed to a reduction in the number of functional groups on GrO. This reduction occurs due to GrO’s interactions with silver ions and the oxygenated structures of cellulose acetate. As a result, tighter networks with smaller pore diameters are formed, leading to slightly reduced permeation compared to the cellulose acetate membrane.

These findings contrast with studies that report an increase in the permeate flow rate of cellulose acetate membranes with the addition of silver [58,64,65]. A key difference in our case is that we used the metal itself as the source of silver nanoparticles, rather than silver nitrate. This approach consumed a greater number of oxygenated functional groups available on graphene oxide for silver aggregation. A decrease in the permeate flow rate was also noted by Jose et al., who demonstrated that the presence of silver in the GrO/AgNP assembly leads to a reduced permeate flow rate compared to membranes containing only GrO. This phenomenon can be explained by the obstruction of the flow path caused by the silver nanoparticles [66,67]. For each cycle, six measurements were taken using the same type of membrane to determine the degree of error between the results, using standard deviation as a statistical calculation method. The largest error observed was ±21.4 L/m^2^h for the CA membrane, while the CA–GrO/AgNP membrane had an error of ±16.3 L/m^2^h.

The flow rates observed for the CA membrane align with those reported by previous studies [38,66], while the CA–GrO/AgNP permeate flow rate falls below the minimum limit recorded in other tests.

### 3.9. Membrane Efficiency

**Figure 9:**
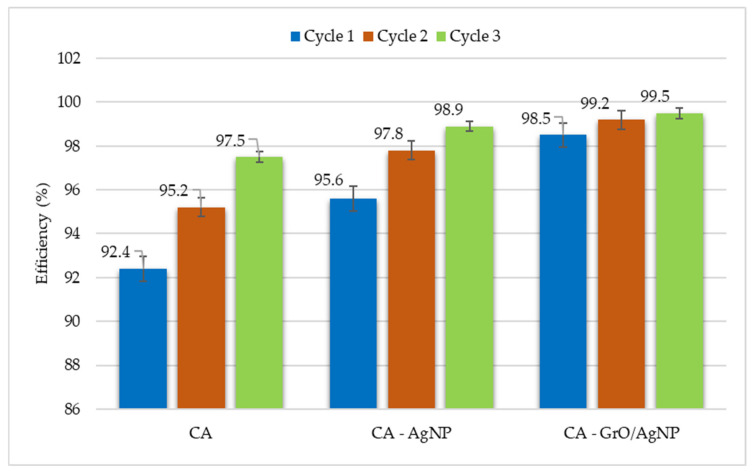
Variation in hydrocarbon removal efficiency.

The data presented in Figure 9 indicate that the efficiency of hydrocarbon removal from the water stream is influenced by the number of passes through the membrane. The CA membrane achieves an efficiency of 97.5% after the third cycle. The performance of the CA–AgNP membrane is moderate, while the CA–GrO/AgNP assembly demonstrates the highest performance, reaching an efficiency of 99.49% by the end of the third cycle. At the end of each cycle, the active surface of the membrane was washed to eliminate polarized hydrocarbons at the interface between the contaminated water and the filtration medium. This result suggests that there are no irregularities in the membrane structure that could lead to preferential flow. Additionally, the compact structure of the membrane indicates that the voids in the CA matrix are filled with decorated nanoparticles, which are immobilized by the functional groups of GrO, contributing to its superior performance. Research on silver-decorated GrO nanoparticles within membrane matrices has shown that this enhanced assembly improves the removal of dyes. Specifically, the removal efficiency of methylene blue increases by 30% when using the GrO/AgNP assembly compared to using graphene oxide alone [35]. This trend of improved efficiency is also supported by the findings in the present study. Tests indicate that, during the initial pass of water contaminated with a 2% heavy oil fraction, the membrane containing the decorated nanoparticles exhibits a 3.9% increase in efficiency compared to the cellulose acetate (CA) membrane that only contains silver nanoparticles. Conducting six consecutive tests with the same type of membrane for each cycle showed a maximum error of ±0.56% for the CA membrane. The calculation of errors was done using the standard deviation statistical function.

## 4. Conclusions

In this study, simple CA membranes and those with a mixed matrix were comparatively analyzed, for which silver nanoparticles and graphene oxide nanoparticles decorated with silver nanoparticles were used. For the synthesis of graphene oxide nanosheets, the Tour method was used, followed by decoration through reduction in the presence of DMF, which facilitated the aggregation of AgNPs.

The membranes were prepared using phase inversion, ensuring that each recipe contained equal weights of nanoparticles. The impact of the decorated structures on the polymer matrix was identified. FTIR analysis revealed a shift of the absorption band corresponding to carbonyl groups towards shorter wavelengths. This shift indicates the formation of electrostatic interactions between the C=O groups and graphene oxide. Additionally, DSC analysis showed a reduction in the intensity of the endothermic peaks, which is characteristic of CA and CA–AgNP. This trend is attributed to the hydrogen bonds formed between the functional groups of CA and GrO, resulting in enhanced stability. Practically, these improvements increase the temperature range in which the mixed matrix membrane can be used without the elution of decorated nanoparticles after many cycles of use. SEM analysis demonstrated the formation of ribs on the membrane’s separation surface due to the concentration of particles. Additionally, silver nanoparticles at the nanometer scale tend to agglomerate, a behavior noted after the surface analysis of decorated nanoparticles.

To evaluate the practical applicability of the synthesized membranes, filterability tests were conducted to determine the permeate flow rates and their efficiency in relation to hydrocarbons. All three membranes were analyzed under identical conditions, with a petroleum fraction rich in olefinic and aromatic structures selected as the contaminant, which can be easily identified through UV analysis. The results indicated that the CA–GrO/AgNP assembly exhibited the lowest permeate flow rates, resulting in a reduction of approximately 160 m^2^/h in flow rate after the first filtration cycle, compared to the simple membrane. However, this reduction was accompanied by an increase in contaminant removal efficiency. After the third cycle, the removal efficiency was 1.99% higher than that of the CA membrane and 0.59% higher than that of the CA–AgNP membrane. To avoid errors in determining efficiency caused by pollutant deposits on the membrane’s active surface, it was washed after each use cycle.

In conclusion, the CA–GrO/AgNP membrane demonstrates significant potential for effectively removing hydrocarbons from wastewater streams. Nevertheless, further studies are necessary to investigate the photocatalytic changes that silver undergoes when deposited on the surface of graphene oxide nanosheets. Additionally, it is important to establish the conditions under which these decorated nanostructures can effectively perform their antimicrobial and antifouling functions when integrated into CA membranes. Furthermore, adjustments can be made to enhance the permeate flow rates, ensuring that the membrane operates efficiently under low driving forces.

## Figures and Tables

**Table 1:** Mass of each element in the composition of membranes.

Membrane Type	CA (g)	AgNP (g)	GrO/AgNP (g)	DMF (mL)
CA	0.75	-	-	5
CA, AgNP	0.75	0.1	-	5
CA, GrO/AgNP	0.75	-	0.1	5

## Data Availability

The original contributions presented in this study are included in the article. Further inquiries can be directed to the main author.

## References

[1] Shi X., Mao D., Song K., Xiang H., Li S., Wang Z. (2024). Effects of Landscape Changes on Water Quality: A Global Meta-Analysis. Water Res..

[2] Ighalo J.O., Pow-Seng Y., Kingsley O.I., Chukwunonso O.A., Tianqi L., Kanika D., Felicitas U.I., Selvasembian R. (2022). Adsorption of Persistent Organic Pollutants (POPs) from the Aqueous Environment by Nano-Adsorbents: A Review. Environ. Res..

[3] Ahmad N.A., Goh P.S.M., Zulhairun A.K., Ismail A.F. (2020). Antifouling Property of Oppositely Charged Titania Nanosheet Assembled on Thin Film Composite Reverse Osmosis Membrane for Highly Concentrated Oily Saline Water Treatment. Membranes.

[4] Husaini I.S., Haddabi M.H. (2025). Recent advances in functionalized electrospun nanofiber membranes for enhanced oily water treatment. J. Environ. Chem. Eng..

[5] Wu Y., Zhu X., Miao D., Song J., Fu S., Zhang J. (2024). Faveolate cell-engineered and multi-heterostructured flexible ceramic nanofibrous membranes with confined coalescent nanochannels enable sustainable oily water remediation. Chem. Eng. J..

[6] Zhu M., Huang C., Mao Y. (2025). Silica-Nanocoated Membranes with Enhanced Stability and Antifouling Performance for Oil-Water Emulsion Separation. Membranes.

[7] Tang Q., Xia L., Liu L., An X., Lan H., Liu H., Qu J. (2025). Polymeric loose nanofiltration membranes based on water-soluble carbon nitride for efficient organics/salts separation. J. Membr. Sci..

[8] Chen J., Ren J., Yang C., Feng B., Dang X., Gong Y. (2025). Enhanced antifouling durability of zwitterionic polymer brush grafted ceramic membrane for sustainable oil/water separation applications. Sep. Purif. Technol..

[9] Liu Y., Du X., Qu Y., Jia H., Xu S., Zhang M. (2025). Fabrication of smart spherical three-dimensional covalent organic framework membranes with switchable oil–water separation via interfacial polymerization. Sep. Purif. Technol..

[10] Wang Y., Chen Z., Zhu Y., Wang H., Cui Z., Li X., Mo J., Li J. (2025). An ultrathin Al_2_O_3_ ceramic membrane prepared by organic-inorganic blending with solvent evaporation and high-temperature sintering for highly efficient oil/water separation. J. Water Process Eng..

[11] Kayanja O., Abdel-Aty A.A.R., Hassan M.A., Hassanin A., Ohashi H., Khalil A.S.G. (2023). A review on TMDCs nanomaterials and their surface engineered polymeric membrane nanocomposites for water remediation and wastewater treatment. Surf. Interfaces.

[12] Ma G., Xu X., Tesfai M., Wang H., Xu P. (2020). Developing anti-biofouling and energy-efficient cation-exchange membranes using conductive polymers and nanomaterials. J. Membr. Sci..

[13] Liu S., Zhang D., Chen W., Wang X., Ji H., Fu Y., Lü C. (2023). Synthesis, antibacterial activity and action mechanism of silver-based nanomaterials with thermosensitive polymer-decorated graphene oxide as a stable support. Mater. Today Commun..

[14] Băjan M., Cursaru D.L. (2025). Study regarding the compatibility between cellulose acetate (CA) and ZnO nanoparticles in polymeric membrane structures using functionalized mphenylenediamine. Dig. J. Nanomater. Biostructures.

[15] Li B., Ansari A.A., Parchur A.K., Lv R. (2024). Exploring the influence of polymeric and non-polymeric materials in synthesis and functionalization of luminescent lanthanide nanomaterials. Coord. Chem. Rev..

[16] Kima N., Lib X., Kimb S.H., Kima J. (2018). Colloidally stable organic–inorganic hybrid nanoparticles prepared using alkoxysilane-functionalized amphiphilic polymer precursors and mechanical properties of their cured coating film. J. Ind. Eng. Chem..

[17] Hao M., Tang M., Wang W., Tian M., Zhang L., Lu Y. (2016). Silver-nanoparticle-decorated multiwalled carbon nanotubes prepared by poly(dopamine) functionalization and ultraviolet irradiation. Compos. Part B.

[18] Schiavi S., Taglietti A., Magni A., Galinetto P., Albini B. (2025). Increasing SERS performance of silver nanoparticles with nanometric coating of polydopamine: A novel approach for methylene blue detection. Appl. Surf. Sci..

[19] Huisman K.T., Abdellah M.H., Sosa D.S.A., Simoes F.R.F., Blankert B., Vrouwenvelder J.S., Szekely G. (2024). Improved cleaning performance of membrane modules using feed spacers modified with cold-plasma treatment and polydopamine and silver-nanoparticle coatings. Desalination.

[20] Sahoo B., Panigrahi L.L., Jha S., Arakha M. (2024). Polyethylene glycol functionalized zinc oxide nanoparticle with excellent photocatalytic performance and oxidative stress mediated bacterial cell death. Opt. Mater..

[21] Akbaria A., Yegania R., Pourabbasb B., Behboudia A. (2017). Analysis of antifouling behavior of high dispersible hydrophilic poly (ethylene glycol)/vinyl functionalized SiO2 nanoparticles embedded polyethylene membrane. Desalination Water Treat..

[22] Choopani L., Mohammadi A., Aliabadi H.A.M., Kashtiaray A., Keihan R.E., Maleki A., Mahdavi M. (2024). Functionalization of zinc ferrites nanoparticles by cyclic aromatic polyimide chains as a novel star polymer with antibacterial activity and low toxicity. J. Ind. Eng. Chem..

[23] Wajahat M., Lee S., Kim J.H., Ahn J., Sim H.H., Kim J.H., Bae J., Seong Hyeon Kim S.H., Pyo J., Seol S.K. (2022). Three-dimensional printing of silver nanoparticle-decorated graphene microarchitectures. Addit. Manuf..

[24] Xin F., Li L. (2011). Decoration of carbon nanotubes with silver nanoparticles for advanced CNT/polymer nanocomposites. Compos. Part A.

[25] Ranjbaran N., Akbari A., Yegani R., Mamaqani H.R., Chapalaghi M. (2025). Graphene oxide decorated copper nanoparticles embedded polysulfone nanocomposite membrane: Anti-bacterial, organo-bio fouling evaluation in pharmaceutical wastewater treatment via MBR. J. Ind. Eng. Chem..

[26] Rahman N., Bharti M., Nasir M., Azmi S.N.H. (2024). Performance assessment of graphene oxide decorated with silver nanoparticles as adsorbent for removal of metformin from water: Equilibrium modeling, kinetic and thermodynamic studies. Next Mater..

[27] Mecha A.C., Chollom M.N., Babatunde B.N., Tetteh E.K., Rathilal S. (2023). Versatile Silver-Nanoparticle-Impregnated Membranes for Water Treatment: A Review. Membranes.

[28] Jung R., Kim Y., Kim H.S., Jin H.J. (2009). Antimicrobial Properties of Hydrated Cellulose Membranes With Silver Nanoparticles. J. Biomater. Sci..

[29] Lkhagvajav N., Koizhaiganova M., Yasa I., Çelik E., Sari Ö. (2015). Characterization and antimicrobial performance of nano silver coatings on leather materials. Braz. J. Microbiol..

[30] Yu Y., Yang Y., Yu L., Koh K.Y., Chen J.P. (2021). Modification of polyvinylidene fluoride membrane by silver nanoparticles-graphene oxide hybrid nanosheet for effective membrane biofouling mitigation. Chemosphere.

[31] Liew K.B., Leong J.X., Daud W.R.W., Ahmad A., Hwang J.J. (2020). Incorporation of silver graphene oxide and graphene oxide nanoparticles in sulfonated polyether ether ketone membrane for power generation in microbial fuel cell. J. Power Sources.

[32] Mahmoudi E., Ng L.Y., Ba-Abbad M.M., Mohammad A.W. (2015). Novel nanohybrid polysulfone membrane embedded with silver nanoparticles on graphene oxide nanoplates. Chem. Eng. J..

[33] Zeeshan M.H., Ruman U.E., Shafiq M., Waqas S., Sabir A. (2024). Intercalation of GO-Ag nanoparticles in cellulose acetate nanofiltration mixed matrix membrane for efficient removal of chromium and cobalt ions from wastewater. J. Environ. Chem. Eng..

[34] Sun X.F., Qin J., Xia P.F., Guo B.B., Yang C.M., Song C., Wang S.G. (2015). Graphene oxide–silver nanoparticle membrane for biofouling control and water purification. Chem. Eng. J..

[35] Song S.H., Kim C.M., Khirul M.A., Ahmad I., Jee H., Chuah C.Y., Park J., Chae K.J., Yang E. (2024). Silver nanoparticle-decorated reduced graphene oxide/nanocrystalline titanium metal-organic frameworks composite membranes with enhanced nanofiltration performance and photocatalytic ability. Desalination Water Treat..

[36] Hoque M.A., Rahman A.F.M.M., Rahman M.M., Bhuiyan M.N.I., Jahan S.A., Shaikh M.A.A., Nurnabi M. (2024). Effect of Successive Recycling and Reuse of Acid Liquor for the Synthesis of Graphene Oxides with Higher Oxygen-to-Carbon Ratios. Heliyon.

[37] Wong-u-ra O., Ekgasit S., Wongravee K. (2017). Phase transferring of silver nanoparticles to organic solvents using modified graphene oxide as carrier. Mater. Chem. Phys..

[38] Alsulaiman L., Abdel-Naby A.S., Alharthi S., Alabdullatif B., Al-Dossary A., Al-Mughrabi W., Alqarni Y. (2025). Synthesis and Characterization of Cellulose Acetate—HBA/Poly Sulfone Blend for Water Treatment Applications. Membranes.

[39] Vanumamalai S.M., Venkatachalam S., Srinivasan N., Dhinakar G. (2025). Optimized hummer’s method for graphene oxide: Structural properties and electrochemical applications. J. Organomet. Chem..

[40] Azman N.H.N., Lim X.C., Sulaiman Y. (2025). Tuning the capacitance of graphene-based materials as negative electrode materials for supercapacitor applications. Diam. Relat. Mater..

[41] Cesaris M.G., Rodríguez M.J.T., Pasan J., Gentili A., Pino V. (2025). Mixed matrix membranes based on cellulose acetate recycled from cigarette butts and metal-organic frameworks for thin film solid-phase microextraction: Determination of phenols in environmental waters. Adv. Sample Prep..

[42] Moraes A.C.M., Andrade P.F., Fariaa A.F., Simões M.B., Salomãoc F.C.C.S., Barros E.B., Goncalves M.C., Alves O.L. (2015). Fabrication of transparent and ultraviolet shielding composite films based on graphene oxide and cellulose acetate. Carbohydr. Polym..

[43] Ahmed D.F., Isawi H., Badway N.A., Elbayaa A.A., Shawky H. (2021). Graphene oxide incorporated cellulose triacetate/cellulose acetate nanocomposite membranes for forward osmosis desalination. Arab. J. Chem..

[44] Chavan V.D., Pinjari D.V., Waghmare N.G., Juikar V.G., Sayyed A.J. (2024). A critical review on technological development of cellulosic material as a sustainable alternative for superabsorbent polymers and its recent applications. Chem. Eng. J..

[45] Volova T.G., Shumilova A.A., Shidlovskiya I.P., Nikolaevab E.D., Sukovatiyb A.G., Vasiliev A.D., Shishatskaya E.I. (2018). Antibacterial properties of films of cellulose composites with silver nanoparticles and antibiotics. Polym. Test..

[46] Teodoro K.B.R., Shimizu F.M., Scagion V.P., Correa D.S. (2019). Ternary nanocomposites based on cellulose nanowhiskers, silver nanoparticles and electrospun nanofibers: Use in an electronic tongue for heavy metal detection. Sens. Actuators B Chem..

[47] Olasupo A., Mohammad R.A.E., Suah F.B.M. (2023). A novel approach in remediation of diclofenac from environmental water samples using silver nanocomposite polymer inclusion membrane. J. Water Process Eng..

[48] Kouda I., Seddik N.B., Boumlasy S.E., Achache M., Zarki Y., Aghmiz A., Tahaikt M., Elmidaoui A., Draoui K. (2025). Impact of solvent treatment on the adsorption efficiency of crystal violet dye using cellulose acetate-clay composite membranes: Experimental and molecular dynamics approaches. Carbohydr. Polym..

[49] Dugam S., Jain R., Dandekar P. (2024). Silver nanoparticles loaded triple-layered cellulose-acetate based multifunctional dressing for wound healing. Int. J. Biol. Macromol..

[50] Benhalima T., Harrar H.F., Doufene N., Sadi A. (2024). Silver decorated zeolite embedded in bionanocomposite hydrogels based on cross-linked carboxymethyl cellulose for excellent catalytic hydrogenation of azo dyes. Int. J. Biol. Macromol..

[51] Kamal T., Ahmada I., Khana S.B., Asir A.M. (2017). Synthesis and catalytic properties of silver nanoparticles supported on porous cellulose acetate sheets and wet-spun fibers. Carbohydr. Polym..

[52] Domke A., Przysiecka L., Jancelewicz M., Jarek M., Coy E., Iatsunskyi I., Richardson J.J., Staszak K., Wozniak-Budych M. (2025). Improving the bioactivity of cellulose acetate hemodialysis membranes through nanosilver modification. Biomater. Adv..

[53] Ali A.S.M., Soliman M.M., Kandil S.H., Ebrahim S., Khalil M. (2022). Tailoring nanocomposite membranes of cellulose acetate/silica nanoparticles for desalination. J. Mater..

[54] Afzal A., Rafique M.S., Iqbal S.S., Butt S.H., Kalsoom U., Rafique M. (2021). Idiosyncratic cellulose acetate nanocomposite membranes: Synthesis and performance control study for desalination. Environ. Technol..

[55] Penkova A., Trchova M., Toikka A.M., Slouf M. (2009). Structure and Pervaporation Properties of Poly(phenylene-iso-phthalamide) Membranes Modified by Fullerene C_60_. Macromol. Mater. Eng..

[56] Elaissaoui I., Sayeb S., Ounif I., Ferhi M., Karima H., Ennigrou D.J. (2024). Preparation and characterization of acetate cellulose electrospun nanofibers membrane: Potential application on wastewater treatment. Heliyon.

[57] Faria A.F., Moraes A.C.M., Andrade P.F., Silva D.S., Goncalves M.C., Alves O.L. (2017). Cellulose acetate membrane embedded with graphene oxidesilver nanocomposites and its ability to suppress microbial proliferation. Cellulose.

[58] Spricka C., Chedea S., Craverb V.O., Escobar I.C. (2018). Bio-inspired immobilization of casein-coated silver nanoparticles on cellulose acetate membranes for biofouling control. J. Environ. Chem. Eng..

[59] Bothun G.D. (2008). Hydrophobic silver nanoparticles trapped in lipid bilayers: Size distribution, bilayer phase behavior, and optical properties. J. Nanobiotechnol..

[60] Bakirdere S., Yilmaz M.T., Tornuk F., Keyf S., Yilmaz A., Sagdic O., Kocabas B. (2015). Molecular characterization of silver–stearate nanoparticles (AgStNPs): A hydrophobic and antimicrobial material against foodborne pathogens. Food Res. Int..

[61] Khalili P., Abdolmaleki A., Riazi M., Davarpanah A. (2025). Enhanced desulfurization Pervaporate via tailored Polypyrrolidone membranes with functionalized graphene oxide nanoparticles and silver ions. Sep. Purif. Technol..

[62] Valenzuela P.G.T., Sánchez A., Isiordia G.E.D., Ontiveros M.M.A., Macias M.R.M., Sicairos S.P., Duarte R.G.S., Weihs G.A.F. (2023). Modifcation and characterization of TFC membranes with Ag nanoparticles: Application in seawater desalination. Polym. Bull..

[63] Hu Y., Jiang J., Dong J., Wang M., Liu S., Tang H., Hong R., He Q., Wada M., Teng B. (2025). Highly flexible free-standing bacterial cellulose-based filter membrane with tunable wettability for high-performance water purification. Int. J. Biol. Macromol..

[64] Yuksel M.S., Tas B., Imer D.Y.K., Koyuncu I. (2014). Effect of silver nanoparticle (AgNP) location in nanocomposite membrane matrix fabricated with different polymer type on antibacterial mechanism. Desalination.

[65] Wang R., Li N., Liu H., Li R., Zhang L., Liu S., Peng Q., Ren L., Liu J., Li B. (2024). Construction of cellulose acetate-based composite nanofiber films with effective antibacterial and filtration properties. Int. J. Biol. Macromol..

[66] Vatanpour V., Pasaoglu M.E., Barzegar H., Teber O.O., Kaya R., Bastug M., Khataee A., Koyuncu I. (2022). Cellulose acetate in fabrication of polymeric membranes: A review. Chemosphere.

[67] Jose J.K., Mishra B., Kootery K.P., Cherian C.T., Tripathi B.T., Sarojini S., Balachand M. (2023). Fabrication of silver nanoparticle decorated graphene oxide membranes for water purification, antifouling and antibacterial applications. Mater. Sci. Eng. B.

